# Pemphigoid Gestationis

**DOI:** 10.5811/cpcem.2018.11.39258

**Published:** 2019-01-07

**Authors:** Rene M. Kukkamalla, Patricia Bayless

**Affiliations:** * Maricopa Medical Center, Department of Emergency Medicine, Phoenix, Arizona; † University of Arizona College of Medicine-Phoenix, Department of Emergency Medicine, Phoenix, Arizona

## CASE PRESENTATION

**Image 1:**
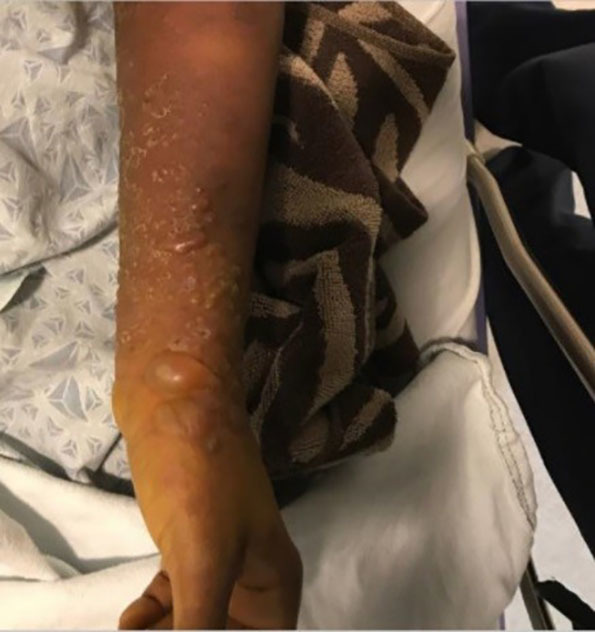
Upper extremity with vesiculobullous lesions in a patient with pemphigoid gestationis.

**Image 2:**
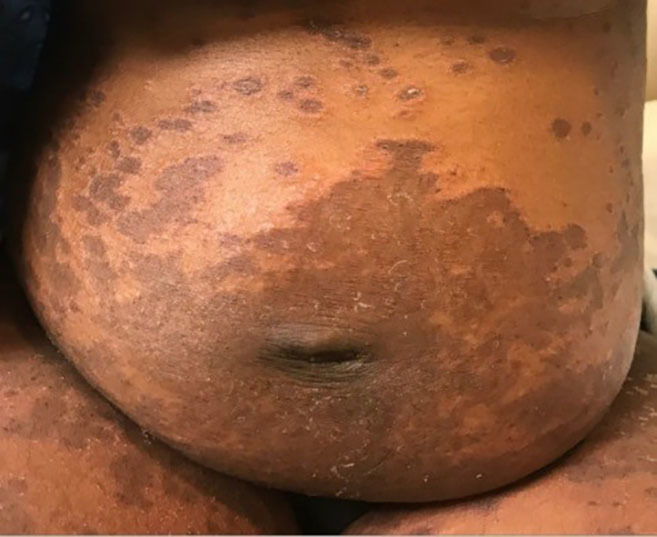
Abdomen with macular lesions in a patient with pemphigoid gestationis.

A 32-year-old female gravida 3 para 2 presented to the emergency department (ED) with two weeks of hyperpigmented macular and blistering rash involving bilateral upper and lower extremities (Image 1) and trunk (Image 2). The patient was approximately 16 weeks pregnant at time of presentation. The rash was significantly pruritic. She denied constitutional symptoms or mucous membrane involvement. The patient was seen by obstetrics/gynecology consult who deemed her rash consistent with pemphigoid gestationis (PG). She was started on high-dose steroid therapy with improvement in the rash. ELISA (an enzyme-linked immunosorbent assay that measures autoantibody reactions to the bullous pemphigoid antigen [BP180] with 96% sensitivity and specificity for PG) showed our patient’s values elevated at 30.59 units (reference range less than 9.0 units).^1^ By the 35th week of pregnancy, her rash had resolved while on a stringent steroid regimen. She developed gestational diabetes that is being managed by her prenatal care provider.

## DISCUSSION

PG is considered a rare disease with estimated incidence approximately 1 in 50,000 pregnancies.^2^ The disease shows a worldwide distribution and no differences in ethnicity. General population studies on the epidemiology of PG are small.^4^ The rash is characterized by pruritic papular and vesiculobullous eruptions that typically involve the abdomen and extremities. The face is classically spared.^2^ It is thought to be an autoimmune phenomenon caused by immunoglobulin G antibody to a 180-kilodaltons antigen in the basement membrane.^1^ Onset of the rash is typically in the second or third trimester and can occur immediately post-partum.^3^

It is important to distinguish this disease from other pregnancy-associated dermatoses because of the high rate of recurrence with more severe symptoms in later pregnancies.^1^,^4^ Typically fetuses are unaffected; however, in less than 5% of cases they can contract transient skin lesions. While fetal outcome is generally good, there is increased risk for intrauterine growth restriction as well as preterm labor.^2^,^4^ Diagnosis is made either clinically, by skin biopsy with direct immunofluorescence (showing linear deposits of complement along the basement membrane), or elevated BP180 antibody levels. Treatment includes topical steroids. For more severe cases systemic steroids are appropriate.^3^, ^4^

**CPC-EM Capsule**

What do we already know about this clinical entity?

*Pemphigoid gestationis (PG) is well described but rarely encountered*.

What is the major impact of the image(s)?

*The images demonstrate a true “pemphigoid” rash*.

How might this improve emergency medicine practice?

*Recognition of PG impacts referral and management of the current and future pregnancies*.
